# Thiamine and selected thiamine antivitamins — biological activity and methods of synthesis

**DOI:** 10.1042/BSR20171148

**Published:** 2018-01-10

**Authors:** Adam Tylicki, Zenon Łotowski, Magdalena Siemieniuk, Artur Ratkiewicz

**Affiliations:** 1 Department of Cytobiochemistry, Faculty of Biology and Chemistry, University of Bialystok, Ciolkowskiego 1J, 15-245 Białystok, Poland; 2 Department of Natural Product Chemistry, Faculty of Biology and Chemistry, University of Bialystok, Ciolkowskiego 1K, 15-245 Białystok, Poland; 3 Department of Theoretical Chemistry, Faculty of Biology and Chemistry, University of Bialystok, Ciolkowskiego 1K, 15-245 Białystok, Poland

**Keywords:** 3-deazathiamine, amprolium, oxythiamine, pyrithiamine, thiamine

## Abstract

Thiamine plays a very important coenzymatic and non-coenzymatic role in the regulation of basic metabolism. Thiamine diphosphate is a coenzyme of many enzymes, most of which occur in prokaryotes. Pyruvate dehydrogenase and 2-oxoglutarate dehydrogenase complexes as well as transketolase are the examples of thiamine-dependent enzymes present in eukaryotes, including human. Therefore, thiamine is considered as drug or diet supplement which can support the treatment of many pathologies including neurodegenerative and vascular system diseases. On the other hand, thiamine antivitamins, which can interact with thiamine-dependent enzymes impeding their native functions, thiamine transport into the cells or a thiamine diphosphate synthesis, are good propose to drug design. The development of organic chemistry in the last century allowed the synthesis of various thiamine antimetabolites such as amprolium, pyrithiamine, oxythiamine, or 3-deazathiamine. Results of biochemical and theoretical chemistry research show that affinity to thiamine diphosphate-dependent enzymes of these synthetic molecules exceeds the affinity of native coenzyme. Therefore, some of them have already been used in the treatment of coccidiosis (amprolium), other are extensively studied as cytostatics in the treatment of cancer or fungal infections (oxythiamine and pyrithiamine). This review summarizes the current knowledge concerning the synthesis and mechanisms of action of selected thiamine antivitamins and indicates the potential of their practical use.

## Introduction

**Figure 1:**
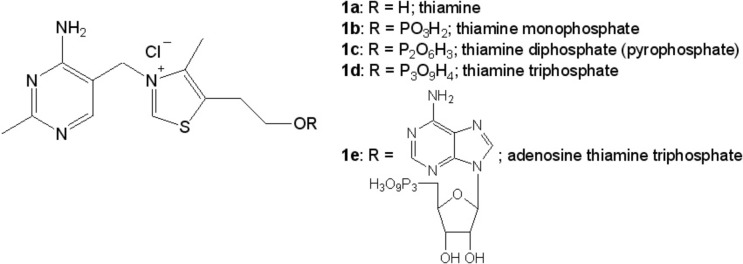
Thiamine and its phosphate derivatives.

All living cells and organisms require many organic compounds to sustain metabolic reactions. One of these compounds is vitamins. Plants, microorganisms, and fungi can synthesize them *de novo*, but many vertebrates, including humans, must supply vitamins with food. Thiamine, vitamin B1 (**1a**; Figure 1) is one of the most important vitamins for maintaining proper functions of most living organisms with individual exeptions among prokaryotes such as *Borrelia burgdorferi* [1]. Thiamine molecule is composed of pyrimidine (4-amino-2-methylpyrimidine) and thiazolium (4-methyl-5-(2-hydroxyethyl)-thiazolium) rings which are linked by a methylene bridge between C3 carbon atom of pyrimidine ring and N3 nitrogen atom of thiazolium ring [2,3].

Identification of the factors which led to the *polyneuritis* (beriberi) contributed to the isolation, determination of chemical structure and method of *in vitro* synthesis of thiamine. In 1897 Dutch medicine doctor Christiaan Eijkman working on the basis of the beriberi treatment stated that rice bran contains the factor, which can reverse the disease symptoms. Christiaan Eijkman has been awarded the Nobel Prize in 1929 for his achievements. The first attempt of isolation of thiamine was carried out at the beginning of the twentieth century. Between the years 1911 and 1912 Polish biochemist Casimir Funk working in Lister Institute in London, isolated from rice bran a substance that counteracts the symptoms of beriberi. Casimir Funk found that the substance contained an amino group, therefore he proposed the name “vitamin”—an amine of life. In 1926 the German biochemists Barend Coenraad Petrus Jansen and Willem Frederik Donath obtained partially purified preparation, which prevented beriberi symptoms in a daily dose of 50 mg. They called this substance aneurine. Unfortunately, Jansen and Donath could not determine the correct structural formula of aneurine. Robert Runnels Williams in the years 1933–1936 clarified the chemical structure of thiamine and developed a method for its synthesis beyond living organism. He also proposed the name “thiamine”, reflecting the presence of both sulfur and the amino group in the molecule [4].

Providing relevant doses of thiamine, in connection with its participation in the metabolism of carbohydrates and bioenergetics processes, are particularly important for the proper functioning of nervous, cardiovascular, and locomotive systems [5–8]. On the other hand, thiamine nutrition is also very important for cancer cells development [9].

Currently, thiamine deficiency is rarely observed in highly developed societies because of diverse diet and wide availability of dietary supplements including vitamins. However, hypovitaminosis B1 may occur in cases of dietary deficiencies or as an effect of certain diseases or excessive use of some drugs (such as furosemide) as well as alcohol abuse [10–13]. Thiamine deficiency are also related with neurodegenerative diseases [7,14–16]. In highly developed societies risk of thiamine deficiency include the elderly, patients after major surgery, pregnant and breastfeeding women, smokers, diabetes, and youth persons prefers high carbohydrate diet [17]. There are data indicating that some populations are especially exposed to thiamine deficiency. For example, mean thiamine diphosphate (**1c**; Figure 1) level in blood serum of control Cambodian mothers was 57 nmol/l while control level in American mothers was 126 nmol/l [18]. In clinical practice, it is recommended to prevent thiamine deficiency by the administration of not more than 30 mg of thiamine hydrochloride daily.

Reduced blood levels of thiamine in case of alcohol addicted people are likely to be the result of their poor diet. Therefore, supplementation of thiamine in alcoholics could prevent of Wernicke–Korsakoff syndrome [19,20]. Clinical observations indicate that similar to Wernicke–Korsakoff syndrome symptoms could appear after surgery (e.g. sleeve gastrectomy and bariatric surgery [21,22]). Thiamine deficiency may also affect 50% of pregnant women. In the light of current research results the hypothesis that maternal thiamine deficiency during pregnancy could cause damage related to child cognitive development should be considered [23]. Thiamine nutritional status has been hypothesized to play an important role in mental health. Research on Chinese adults (50–70 years old) showed the correlation between thiamine and its derivative’s concentration in the blood and depression symptoms [24].

Thiamine nutrition is a serious problem in geriatrics. Research carried out on patients aged 76–90 years showed the state of hypovitaminosis B_1_ in more than 40% and 20% of hospitalized and ambulatory patients respectively [25]. In these groups of patients thiamine deficiency was associated with diuretics administration, unbalanced diet as well as the reduction in the rate of thiamine absorption in the digestive system [26]. These data indicate that controlled thiamine supplementation can significantly improve the quality of life of elderly people and can reduce the possibility of dementia [7,15].

There is a lot of premises suggesting connection of thiamine metabolism with carcinogenesis. However, the relationship between vitamin B_1_ and initiation as well as development of cancer still remains unknown. Some authors postulate that thiamine increases cancer cell’s viability and survival and it is involved in increase in cancer cell’s resistance to therapy [27]. Other data indicate that reduced thiamine level increases risk of some kinds of cancer development [28]. On the other hand, there are evidence that thiamine can protect from tumors of central nervous system [29]. The effect of thiamine supplementation on cancer cell depends on thiamine dose—low doses stimulate whereas high doses inhibit cancer cell growth. The first effect is probably from coverage of energy demand and increased synthesis of essential nutrients which are fundamental to intensively dividing cancer cells. The effect of cancer inhibition is explained by inactivation of pyruvate dehydrogenase kinases by high level of thiamine diphosphate. During carcinogenesis, cells inactivate pyruvate dehydrogenase complex through phosphorylation by overexpression of pyruvate dehydrogenase kinases. Inhibition of kinases by thiamine diphosphate revers this effect and maintains pyruvate dehydrogenase complex activity on normal level. [30].

**Figure 2:**
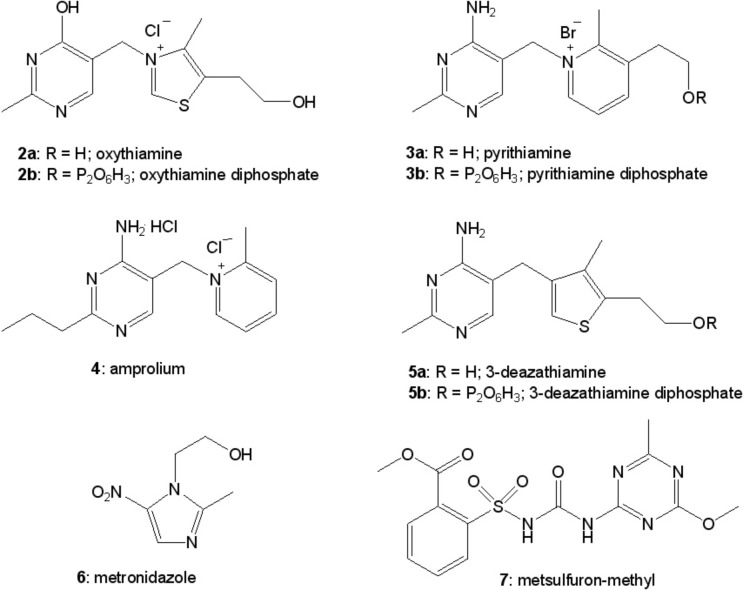
Selected synthetic antivitamins of thiamine.

Taking into consideration all those facts, thiamine still represents as a valuable drug and a dietary supplement in many studies taken by biologists, medics, and chemists. On the other hand, synthesis of its antagonists (e.g. oxythiamine (**2a**; Figure 2), pyrithiamine (**3a**; Figure 2), amprolium (**4**; Figure 2), 3-deazathiamine (**5a**; Figure 2)) was of great importance in understanding of vitamin B1 metabolic role and consequences of avitaminosis. In the last 50 years, a lot of data regarding various thiamine analogs have appeared [31–35]. Depending on the nature of the modification, synthetic thiamine derivatives may be biologically inactive and act as antivitamins. Despite many years of research, even recently some of the known derivatives of thiamine were used in research to induce experimental conditions similar to the thiamine deficiency in animal models (pyrithiamine [36,37]) and during the study of functioning of thiamine diphosphate-dependent enzymes and regulation of expression of genes involved in thiamine synthesis (oxythiamine [38,39]). Some of these derivatives are used in medicine to treat bacterial infections (metronidazole (**6**; Figure 2) [31]), in veterinary to treat the parasites infections (amprolium [6,40]), and in agriculture as herbicides (metsulfuron-methyl (**7**; Figure 2), [41,42]). The results of recent research show the perspective of usage of thiamine analogs as cytostatics in cancer treatment [32,43] and fungal infections [44].

This article summarizes achievements in the field of chemical synthesis and understanding of biological activity of selected antimetabolites of vitamin B1 in the light of thiamine role in the basic metabolic processes of the cells.

## The role of thiamine in the basic metabolic pathways of the cell

In living organisms, thiamine is present in a free form, and as its phosphorylated derivatives: thiamine monophosphate (**1b**; Figure 1), diphosphate (**1c**; Figure 1), triphosphate (**1d**; Figure 1), and adenosine thiamine triphosphate (**1e**; Figure 1). Thiamine diphosphate in cells occurs in the largest concentrations (70–90% of total thiamine and its derivatives). The total concentration of thiamine and its derivatives in the blood of animals is approximately 1 μM, while in humans only 0.1 μM. In rat brain, thiamine and its derivatives are present in concentrations of 6–13 nmol/g wet weight, while in humans are only 3–4 nmol/g [45,46]. These data indicate that humans are strongly exposed for vitamin B1 deficiency and therefore emphasize the importance of proper regulation of thiamine-dependent processes in cell metabolism.

The role of thiamine and its derivatives in the metabolism of the cells can be considered in three aspects. First, as a cofactor of many enzymes which control bioenergetic [47–50], amino acid metabolism [51], and transformation of various carbohydrates, including pentoses, necessary for the synthesis of nucleotides [52]. Second, we cannot underestimate the non-coenzymatic role of phosphorylated derivatives of thiamine in control of cell metabolism by: allosteric regulation of enzymes [53,47–49], transmission of nerve signals in synapses and likely involved in signaling pathways associated with receiving stimuli from the environment [54,55], and regulation of protein synthesis by so-called riboswitches in microorganisms and plants [56,57]. Finally, the results of many studies strongly suggest that thiamine, its phosphorylated derivatives, and thiamine-dependent enzymes play an important role in the reaction of microorganisms [58,59], animals [60,61], and plants [2,62] on various environmental factors like oxidative stress and pathogens.

**Figure 3:**
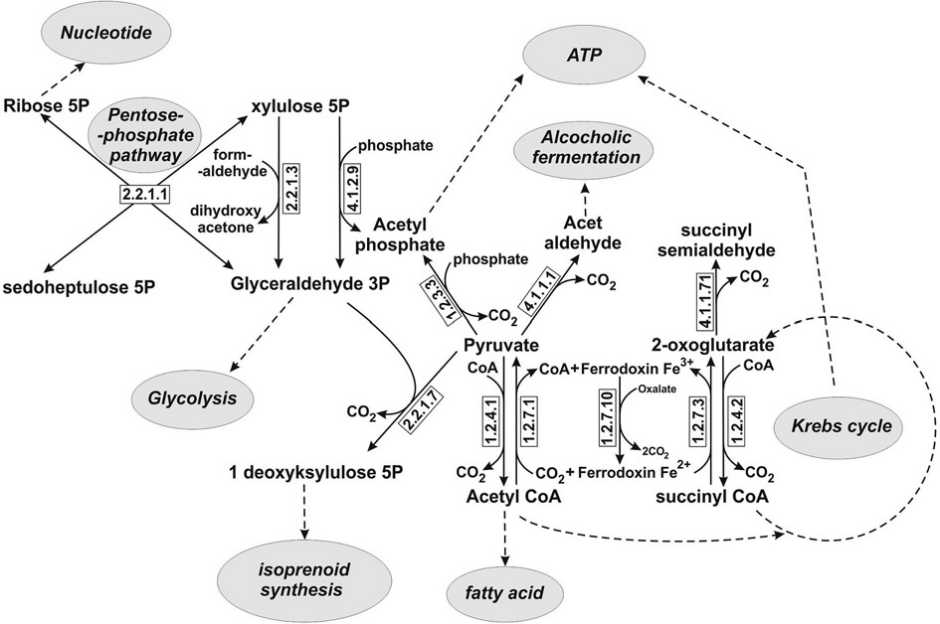
Main metabolic reactions catalyzed by thiamine pyrophosphate-dependent enzymes in prokaryotic cells Continuous lines represent reactions catalyzed by thiamin pyrophosphate-dependent enzymes whereas dashed lines represent processes which are indirectly linked with thiamin pyrophosphate-dependent enzymes. Symbols above the arrow specify EC numbers of individual enzymes: 1.2.7.3, 2-oxoglutarate ferrodoxin oxidoreductase; 1.2.4.2, 2-oxoglutarate dehydrogenase (component E1 of 2-oxoglutarate dehydrogenase complex; 1.2.7.10, oxalate oxidoreductase; 1.2.7.1, pyruvate ferrodoxin oxidoreductase; 1.2.4.1, pyruvate dehydrogenase (component E1 of pyruvate dehydrogenase complex); 1.2.3.3, pyruvate oxidase; 2.2.1.3, dihydroxyacetone synthase; 2.2.1.1, transketolase; 2.2.1.7, 1-deoxy-D-xylulose 5-phosphate synthase; 4.1.2.9, phosphoketolase; 4.1.1.1, pyruvate decarboxylase; 4.1.1.71, indolepyruvate decarboxylase.

Thiamine after phosphorylation to thiamine diphosphate acts as a coenzyme of many enzymes catalyzing various carboxylation and decarboxylation reactions, as well as reversible transfer of two-carbon fragments between various donors and acceptors. Deprotonation of C2 carbon atom in the thiazolium ring and formation of ylide are the basis of thiamine diphosphate-dependent reactions [63,64]. Recent research indicates that amino group of pyrimidine ring also plays an essential role in decarboxylation process [38]. The key biochemical pathways including synthesis and degradation of carbohydrates, amino acids, and nucleotides involve thiamine diphosphate-dependent enzymes. There is a large database concern known protein sequence and structure of thiamine diphosphate-dependent enzymes [65]. There are many thiamine diphosphate-dependent enzymes and all of them occur in almost every living organism with the exception of some prokaryotes such as *Borrelia burgdorferi* and cyanobacteria. *B. burgdorferi* has no genes encoding thiamine transporters, thiamine biosynthesis enzymes, and thiamine diphosphate-dependent enzymes as well [1], whereas cyanobacteria have no 2-oxoglutarate dehydrogenase [66]. Only few of these enzymes occur in human cells [32]. In many prokaryotes and other microorganisms including yeast thiamine diphosphate-dependent reactions are involved in bioenergetics (alcoholic fermentation, oxidative phosphorylation, and substrate level phosphorylation) and many anabolic reactions like photosynthesis, fatty acid, isoprenoid, and nucleotide biosynthesis (Figure 3).

Very important thiamine diphosphate-dependent enzyme for human economy is pyruvate decarboxylase which occurs in organisms obtaining energy by alcoholic fermentation. Pyruvate decarboxylase is relatively widespread in plants, fungi, and bacteria [67]. The enzyme catalyzes irreversible reaction of pyruvate decarboxylation to acetaldehyde. This is the first step in the production of ethanol, which is subsequently synthetized by reduction of acetaldehyde by alcohol dehydrogenase. This reaction, utilizing substrate provided by pyruvate decarboxylase, is one of the most efficient ways to supply an oxidized NAD^+^ necessary to sustain the glycolysis process. Taking into consideration, the role of ethanol as biofuel and possibility of its production from cellulosic biomass fusion of pyruvate decarboxylase and alcohol dehydrogenase proteins form *Zymomonas mobilis* was generated and expressed in *Escherichia coli*. Cells expressing the fusion protein generated ethanol more rapidly and reached its higher levels [68].

**Figure 4:**
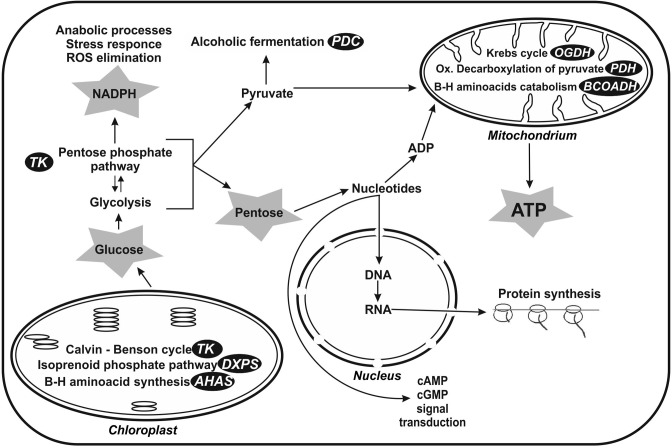
Cell localization of main thiamine diphosphate-dependent enzymes and its participation in metabolic pathways of eukaryotic cells Shortcuts on a black background indicating the mine enzymes: AHAS, acetohydroxyacid synthase; BCOADH, branched chain 2-oxoacids dehydrogenase (E1 component of branched chain 2-oxoacids dehydrogenase complex); DXPS, 1-deoxy-D-xylulose 5-phosphate synthase; OGDH, 2-oxoglutarate dehydrogenase (E1 component of 2-oxoglutarate dehydrogenase complex); PDC, pyruvate decarboxylase; PDH, pyruvate dehydrogenase (E1 component of pyruvate dehydrogenase complex); TK, transketolase. Gray asterisk – metabolites directly associated with thiamine pyrophosphate-dependent pathways. Thiamine diphosphate-dependent enzymes play a role in photosynthesis in chloroplasts (TK, DXPS), pentose phosphate pathway (TK), and alcoholic fermentation (PDC) in cytoplasm as well as in ATP synthesis by participation in oxidative decarboxylation of pyruvate (PDH) and Krebs cycle (OGDH) in mitochondria. These enzymes are also involved in branched amino acid synthesis (AHAS) and catabolism (BCOADH). Pentose phosphate pathway supplies NADPH which is necessary for anabolic processes and reduction of natural antioxidants. Moreover, it provides pentose necessary for nucleotide synthesis.

In eukaryotic cells, thiamine diphosphate-dependent enzymes take part in the most fundamental processes of cellular metabolism [32,63,69] (Figure 4). In the case of animals and humans, the most important thiamine-dependent enzymes are mitochondrial multienzyme complexes of pyruvate- and 2-oxoglutarate dehydrogenases as well as cytoplasmic transketolase.

Pyruvate dehydrogenase complex (PDHC) plays an important role in bioenergetic processes controlling supply of acetyl-CoA into the Krebs cycle and anabolic reactions by linking glycolysis and Krebs cycle through oxidative decarboxylation of pyruvate. The essential role of PDHC in the cell metabolism is manifested in majority of clinical features of its deficiency, for example mental retardation, ataxia, peripheral neuropathy, structural brain abnormalities (cerebral atrophy and ventriculomegaly). Most patients affected by congenital PDHC deficiency die in the first 20 years of life [70]. There are evidence that pathological accumulation of reactive oxygen species in cells is related with PDHC deficiency. Activity of mitochondrial manganese superoxide dismutase is strongly reduced in PDHC-deficient cells [71]. The regulation of PDHC activity is very important in cancer cells. Hypoxia inhibitory factor α1 (HIF1α) activates pyruvate dehydrogenase kinase and inhibits activity of PDHC and stimulates the Wartburg effect [72]. Therefore, PDHC is a good target for tumor therapy. Regulation of PDHC activity is very important during aging and in neurodegenerative diseases. In normal rat brain astrocytes, PDHC is strongly inhibited by high expression of PDH-kinase, whereas neuronal PDHC activity is high because of lower kinase activity [73]. During Alzheimer’s disease, reduced PDHC activity was observed [74]. The results of experiments carried out on *Caenorhabditis elegans* show that its survival was reduced by knockout of pyruvate dehydrogenase. On the other hand, it was enhanced by knockout of pyruvate dehydrogenase kinase [75].

2-Oxoglutarate dehydrogenase complex is one of the main regulatory points of the Krebs cycle. Moreover, it controls a distribution of succinyl-CoA and 2-oxoglutarate for substrate phosphorylation of GDP, ADP, or for synthesis of several amino acids and heme [50]. Recent data indicated that specific responses of cancer cells to 2-oxoglutarate dehydrogenase complex inhibition could be used in cancer diagnosis [76]. Moreover, this enzyme could be a promising target in cancer therapy especially in the case of cancer cells which generate significant quantities of ATP through oxidative metabolism such as breast and cervical cancer. The main source of ATP of cervical cancer is oxidative phosphorylation, which cover approximately 95% of energy requirements. Similarly, breast cancer cell line MCF7 acquires approximately 80% of ATP through oxidative phosphorylation. Oxidative metabolism is also preferred by uterus cancer and HeLa cell lines. In both cases, it was found that 90% production of ATP is realized by oxidative phosphorylation [77].

Transketolase is the main thiamine diphosphate-dependent enzyme of a nonoxidizing branch of the pentose phosphate pathway, which catalyzes the reversible transfer of xylulose-5-phosphate and ribose-5-phosphate to sedoheptulose-7-phosphate and glyceraldehyde-3-phosphate, or erythrose-4-phosphate and xylulose-5-phosphate to fructose-6-phosphate and glyceraldehyde-3-phosphate. Through participation in the pentose phosphate pathway, transketolase has three important functions in the metabolism of the cells. First, it provides pentoses for the synthesis of nucleotides. Second, it can provide metabolites for glycolysis or glukoneogenesis pathway. Third, it has indirect influence on the synthesis of NADPH, required for the anabolic processes and reduction of natural antioxidants (glutathione, ascorbic acid). Therefore, the maintenance of transketolase activity on the appropriate level is essential for the proper functioning of lipids and carbohydrates metabolism, as well as replication process. Numerous studies have implicated the role of transketolase in the pathogenesis of neurodegenerative diseases, diabetes, and cancer [16,43,52,78–82].

Beyond commonly accepted thiamine diphosphate action as a coenzyme of basic metabolic pathways, thiamine has long been known to its non-coenzyme action in brain, particularly in relation to nerve function. Thiamine triphosphate may be involved in nerve impulse transmission acting on the ligand-gated sodium channels and voltage-gated chloride channels [14,83,84]. Moreover, it may functioning as a specific donor of phosphate group in phosphorylation of synaptosomal proteins [55].

Independent studies revealed that thiamine triphosphate can act as a signaling molecule in adaptation of bacteria to stress conditions [84–86]. Adenosine thiamine triphosphate probably also plays a role in response to specific conditions of abiotic stress [87]. Moreover, it is known that adenosine thiamine triphosphate regulates activity of membrane adenosine thiamine triphosphate transporter [88] and poly(ADP-ribose) polymerase-1 (PARP-1) [89].

Taking into account the role of thiamine and thiamine diphosphate-dependent enzymes, the synthesis of thiamine antimetabolites is justified in terms of regulation of cell metabolism as well as their cytostatic potential.

## The synthesis of thiamine and selected thiamine antimetabolites

### The synthesis of thiamine

**Figure 5:**
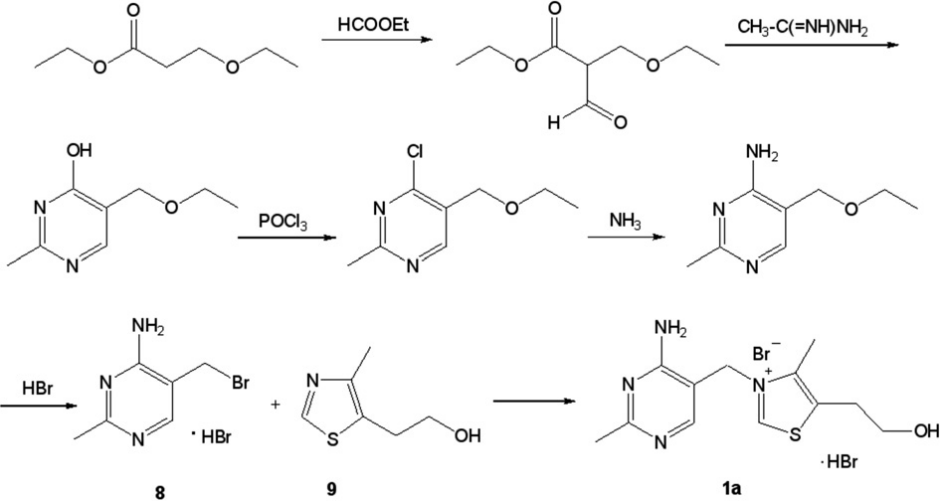
Synthesis of thiamine, method by Williams and Cline [90].

The first synthesis of thiamine (**1a**, Figures 1 and 5) was performed by R. R. Williams and J. K. Cline in 1936 [90] by the route depicted at Figure 5. In the crucial step of this work, 5-(2-hydroxyethyl)-4-methylthiazole (**9**, Figure 5) was subjected to the quaternization reaction with 4-amino-5-ethoxymethyl-2-methylpyrimidine (**8**, Figure 5) to give the expected product (**1a**, Figure 5). This method is still successfully used nowadays, although there have been a lot of publications describing modifications of thiazole ring synthesis (e.g*.* [91]).

### The synthesis of oxythiamine

**Figure 6:**
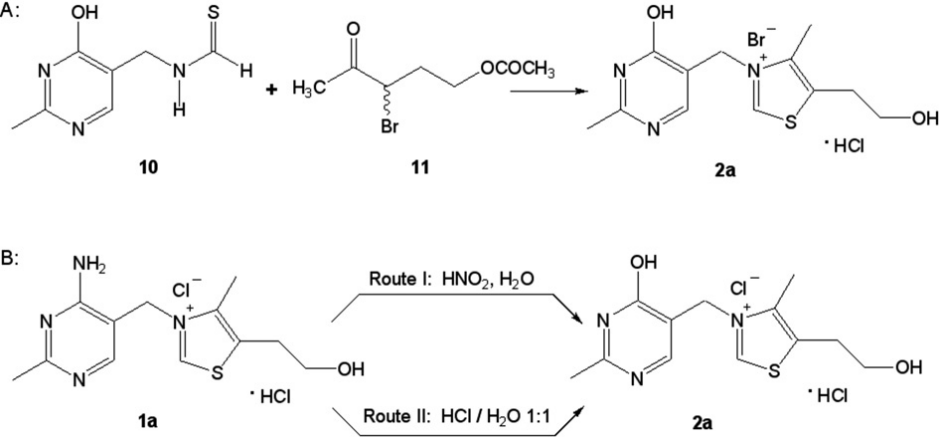
Two methods for synthesis of oxythiamine.

Oxythiamine (**2a**, Figures 2 and 6) was for the first time prepared synthetically by Bergel and Todd [92] as a result of condensation of 4-hydroxy-5-thioformamidomethyl-2-methylpyrimidine (**10**, Figure 6A) with 3ξ-bromo-4-oxopentyl acetate (**11**, Figure 6A), but the procedure was laborious and the final product was obtained with rather moderate yield.

Soodak and Cerecedo [93] converted thiamine (**1a**, Figure 6B) into oxythiamine in 50–70% yield by deamination of the substrate with nitrous acid (Figure 6B, route I).

The highly efficient method for the preparation of oxythiamine, also in large scale, was developed by Rydon [94]. Oxythiamine, essentially free from thiamine, can be prepared in 80% yield by refluxing of the substrate with 5N hydrochloric acid for 6 h (Figure 6B, route II).

### The synthesis of pyrithiamine

**Figure 7:**
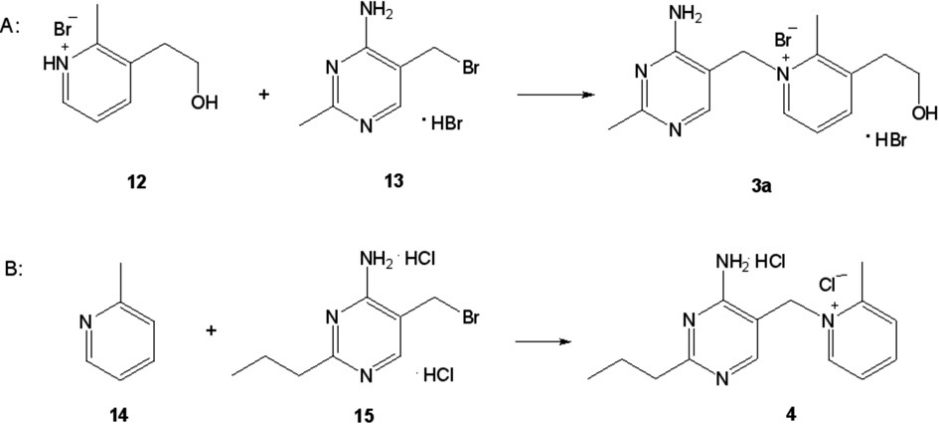
Synthesis of pyrithiamine (**A**) and amprolium (**B**).

For the first time, pyrithiamine (**3a**, Figures 2 and 7) was synthesized by Tracy and Elderfield [95] by quaternization of 3-(2-hydroxyethyl)-2-methylpyridine (**12**, Figure 7A) with 4-amino-5-bromomethyl-2-methylpyrimidine hydrobromide (**13**; Figure 7A). The product gave acceptable elemental analysis for C and H provided that a molecule of water of crystallization was assumed. A substance made according to these protocols was called pyrithiamine and was used to demonstrate that typical signs of thiamine deficiency of animals could be elicited by feeding it [96]. However, Wilson and Harris [97] observed that such material did not give correct analytical values for N. By modifying the temperature and solvent of the condensation and by using an excess of the pyridine component (**12**, Figure 7A), they were able to prepare pure compound which gave correct analytical values for all of its constituent elements. Therefore, they concluded that their material differed in structure from what had been named pyrithiamine, and proposed a new name for this compound— neopyrithiamine. When the biological activity of pyrithiamine was compared with neopyrithiamine, no qualitative difference between them was found. Quantitatively, neopyrithiamine was approximately four times as active. These obvious similarities in biological behavior suggested that the active component of pyrithiamine was probably identical with neopyrithiamine, and pyrithiamine was just impure neopyrithiamine. Moreover, it has been possible to isolate from pyrithiamine a substance with the characteristic UV absorption maxima of neopyrithiamine. All these observations led Woolley [98] to the conclusion that neopyrithiamine is pure pyrithiamine and the substance described by Tracy and Elderfield [95] was heavily contaminated with biologically inert material.

In later years, a number of papers have been published in which the synthetic pathways leading to both pyridine and pyrimidine substrates (**12** and **13**, Figure 7A) have been improved (e.g. [99–101]).

### The synthesis of amprolium

The first synthesis of amprolium (**4**, Figures 2 and 7) was performed by Rogers et al*.* [102–104]. The key step of the work was the quaternization reaction of 2-picoline (**14**, Figure 7B) with 4-amino-5-bromomethyl-2*n*-propylpyrimidine dihydrochloride (**15**; Figure 7B). A new approach for the preparation of amprolium has been presented in the papers [105,106].

### The synthesis of 3-deazathiamine

**Figure 8:**
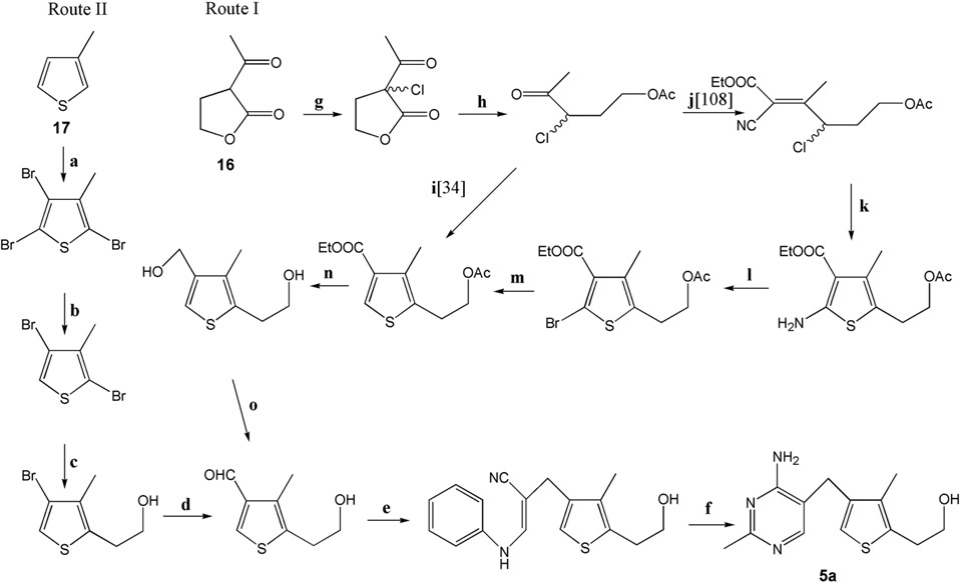
Reagents and reaction conditions of 3-deazathiamine synthesis (**a**) (1) Br_2_, CHCl_3_; (2) KOH/EtOH, (**b**) Zn, AcOH [88] or *s*-BuLi [89], (**c**) *n*-BuLi, ethylene oxide, BF_3_**^.^**Et_2_O, (**d**) *n*-BuLi (2 eq), DMF, (**e**) 3-anilinopropionitrile, NaOMe/MeOH, DMSO, (**f**) acetamidine hydrochloride, NaOEt/EtOH, (**g**) SO_2_Cl_2_, (**h**) AcOH, HCl, Ac_2_O, (**i**) (1) NaHS; (2) ethyl 3-ethoxyacrylate, LiHMDS; (3) HCl, (**j**) NCCH_2_COOEt, AcONH_4_, C_6_H_5_CH_3_, (**k**) NaSH, EtOH, (**l**) CuBr_2_, *t-*BuONO, CH_3_CN, (**m**) Zn, AcOH, (**n**) LiAlH_4_, Et_2_O, (**o**) MnO_2_, CHCl_3_.

In general, two methods for the preparation of 3-deazathiamine (**5a**, Figures 2 and 8) are known. In the first synthetic pathway [34,107] (Figure 8, route I), 2-acetylbutyrolactone (**16**, Figure 8) is applied as a starting material to construct properly substituted thiophene ring. In the next stage, substituted pyrimidine ring is built using 3-anilinopropionitrile and acetamidine hydrochloride. In the second method [108,109] (Figure 8, route II), 3-methylthiophene (**17**, Figure 8) is used as a substrate. The incorporation of suitable functional groups (2-hydroxyethyl and formyl) into its ring is followed by the construction of pyrimidine ring in the same way as in method 1.

### The introduction of diphosphate moiety

The most common method for the phosphorylation of thiamine or related compounds is the reaction between an alcohol and concentrated phosphoric acid at high temperatures (100–140°C) [110,111]. The resulting mixture contains mono-, di-, and triphosphates of the thiamine analog which needs to be separated from each other and the vast excess of inorganic phosphates. There are several known ways to achieve this. Cerecedo et al*.* [112] prepared oxythiamine diphosphate as above and purified it by multiple recrystallization from acetone.

Ban and co-workers [113], in turn, synthesized both pyrithiamine and oxythiamine diphosphates and showed that the mixture of mono-, di-, and triphosphates could easily be separated by the HPLC chromatography.

A procedure for the preparation of the pure crystalline phosphoric esters of oxythiamine (monophospho-oxythiamine, diphospho-oxythiamine, and triphospho-oxythiamine) was described by Navazio et al. [114]. This method is based on the electrophoretic separation of a mixture of oxythiamine phosphoric esters, obtained by chemical phosphorylation of oxythiamine.

A different synthetic route should have been applied to the 3-deazathiamine diphosphate synthesis due to the fact that extremely acidic conditions associated with the use of concentrated phosphoric acid caused the decomposition of the substrate. The alternative method that Leeper and co-workers tried employed S_N_2 displacement of a good leaving group (*p*-toluenesulfonyloxy) by a diphosphate ion derived from tris(tetra-*n*-butylammonium) hydrogen diphosphate [115].

## Biological activity of selected thiamine analogs

Generally, there are three ways of thiamine antimetabolites influence on the cells—inhibition of thiamine diphosphate-dependent enzymes, influence on thiamine uptake, and blocking of thiamine phosphorylation process [31,33,35]. Oxythiamine, pyrithiamine, and 3-deazathiamine after phosphorylation can may be incorporated into active centers of thiamine diphosphate-dependent enzymes causing their inactivation and inhibition of the metabolic pathway, in which these enzymes are involved. Some analogs of thiamine, for example amprolium, are difficult to interact with the active centers of enzymes due to inability of phosphate esters formation. Such derivatives can affect cell metabolism by inhibition of thiamine intake. Thiamine is transported into the cell not only by ThTr1 and ThTr2, but also by organic cation transporters family (most probably OTC1 and OTC3) and amprolium significantly decreases this process [116]. Pyrithiamine, in addition to the impact on thiamine diphosphate-dependent enzymes, may also inhibit thiamine transformation into thiamine diphosphate by inhibition of thiamine pyrophosphokinase.

### Thiamine antimetabolites and thiamine diphosphate-dependent enzymes

Valuable information about the impact of thiamine antivitamins on thiamine diphosphate-dependent enzymes provides results of enzymological *in vitro* experiments. The strength of coenzyme binding in the active center varies depending on the enzyme. Transketolase (TK), 2-oxoglutarate dehydrogenase complex (OGDHC), and pyruvate decarboxylase (PDC) bind coenzyme stronger than PDHC and in case of the first two enzymes it is difficult to obtain apoform. Rat liver TK was inhibited at 50% by oxythiamine diphosphate in concentrations of 0.02–0.2 μM [117]. *I*_50_ value of oxythiamine diphosphate for yeast transketolase was approximately 0.03 μM and even addition of 0.5 μM of thiamine diphosphate did not restore the enzyme activity [118]. It may suggest that affinity of oxythiamine diphosphate to the enzyme is even higher in comparison with natural coenzyme. Investigations of yeast transketolase apoform confirm this hypothesis [119]. In these research, *K*_i_ values for oxythiamine diphosphate (0.03 μM) was lower than *K*_m_ for thiamine diphosphate (1.1 μM) in contrast with pyrithiamine diphosphate (110 μM). Bovine adrenals OGDHC contained 70% apoform was obtain by Taranda et al. [120]. Inhibition of this enzyme activity by oxythiamine diphosphate was competitive and its *K*_i_ values was approximately 30 μM whereas *K*_m_ for thiamine diphosphate was 6.7 μM in presence of Mg^2+^ or 33 μM in presence of Mn^2+^. In this case, anti-coenzyme did not inhibit the holoform of the enzyme. Other data indicate that OGDHC holoform from European bison heart was inhibited by even lower doses of oxythiamine diphosphate [121] (*I*_50_ = 24 μM). Comparison of these data indicates that inhibition effect is tissue- or species-specific and competitive displacement of natural coenzyme by anti-coenzyme may occur.

PDHC binds coenzyme weaker than TK and OGDHC and therefore it is more sensitive to thiamine antivitamins. Kinetic data of PDHC apoform isolated from European bison heart (oxythiamine diphosphate, *K*_i_ = 0.23 μM; thiamine diphosphate, *K*_m_ = 0.6 μM [121]) and bovine adrenals (oxythiamine diphosphate, *K*_i_ = 0.07 μM; thiamine diphosphate, *K*_m_ = 0.11 μM [122]) as well as bovine heart (oxythiamine diphosphate, *K*_i_ = 0.04 μM; thiamine diphosphate, *K*_m_ = 0.07 μM [123]) indicates that *K*_m_ for thiamine diphosphate is often higher than *K*_i_ values for anti-coenzyme. Similar relationship for oxythiamine diphosphate (*K*_i_ = 20 μM) was obtained for PDC from yeast [124] but in the case of pyrithiamine *K*_i_ value was higher (78 μM) in comparison with *K*_m_ for thiamine diphosphate (23 μM). All these results confirm that oxythiamine diphosphate, in contrast with pyrithiamine diphosphate, show a higher affinity to the thiamine diphosphate-dependent enzymes compared with the natural coenzyme.

**Figure 9:**
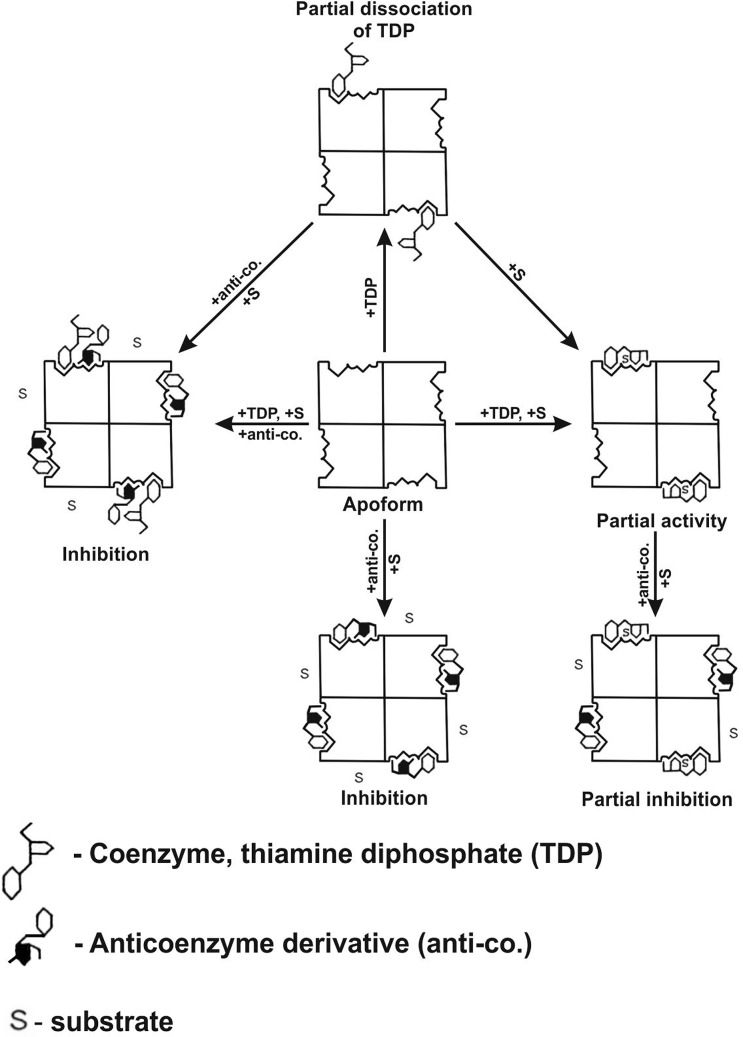
Schematic illustration of the possible functioning of PDHC semisaturated with thiamine pyrophosphate and influence of anti-coenzyme derivatives on enzyme activity Partial dissociation of the endogenous thiamine pyrophosphate in the absence of substrate allows the binding of anti-coenzyme derivative and inhibition of enzyme in the case of semisaturated as well as saturated concentration of coenzyme. Some anti-coenzyme binding often occurs with the same or even greater affinity in comparison with native coenzyme. Addition of substrate to the enzyme with partially dissociated coenzyme caused reassociation of coenzyme and activation of complex. Addition of substrate to the enzyme containing partially dissociated coenzyme and anti-coenzyme did not cause reactivation of enzyme.

It is interesting that some enzyme holoforms (e.g. PDHC which binds thiamine diphosphate weakly) is also sensitive to oxythiamine diphosphate in contrast with other enzymes (e.g. OGDHC and TK which bind the coenzyme stronger). This phenomenon can be explained by hypothesis of partial dissociation of the endogenous thiamine diphosphate in the absence of substrate [125,121]. Kinetic data give some evidence that thiazolium ring and diphosphate moiety of thiamine diphosphate are capable of release from the active site of PDHC in the absence of pyruvate [126]. Incomplete association of thiamine diphosphate in active centers of E1 component of PDHC may allow for anti-coenzyme binding into enzyme even in presence of coenzyme. Formation of such complex impedes the substrate binding and catalysis (Figure 9). The validity of this interesting hypothesis needs to be confirmed by more specific theoretical chemistry and crystallographic research.

There are data that another anti-coenzyme of thiamine diphosphate, 3-deazathiamine diphosphate can bind to target thiamine diphosphate-dependent enzymes with greater affinity and speed than the natural coenzyme. Studies of *Z. mobilis* PDC and the *E. coli* OGDHC suggest that 3-deazathiamine diphosphate binds to these enzymes 25000- and 500-times more tightly than natural coenzyme, respectively (*K*_i_ value versus PDC is 14 pM, and versus OGDHC 5 nM [115]). Moreover authors suggest that 3-deazathiamine, which lacks the diphosphate portion, binds to 2-hydroxy-3-oxoadipate synthase from *Mycobacterium tuberculosis* with affinity similar to thiamine diphosphate, but 3-deazathiamine diphosphate binds 32-fold more tightly to the enzyme than natural coenzyme [109]. Thus, 3-deazathiamine diphosphate can be considered as an exceptional inhibitor among other known, for which *K*_i_ values are usually in the range of hundredths to tens μM [32]. It is very interesting that the lack of nitrogen atom with positive charge in thiazolium ring may increases the affinity of the analog to the active site of enzymes so strongly. Authors suggest that high affinity of this compound to thiamine diphosphate-dependent enzymes is based on hydrophobic interactions of 3-deazathiamine with nonpolar amino acids in enzymes active center. Remarkably, this very potent inhibitor was not investigated in *in vitro* as well as *in vivo* models as a potential cytostatic till now.

**Figure 10:**
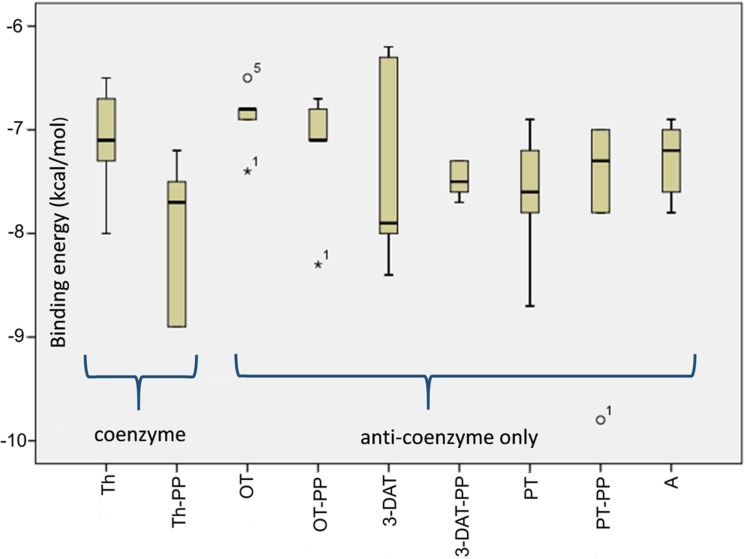
Statistical distribution of binding energies at binding points of thiamine and its derivatives to the pyruvate decarboxylase 3-DAT, 3-deazathiamine; A, amprolium; OT, oxythiamine; PT, pyrithiamine; –PP, diphosphate esters of above mentioned compounds; Th, thiamine.

**Figure 11:**
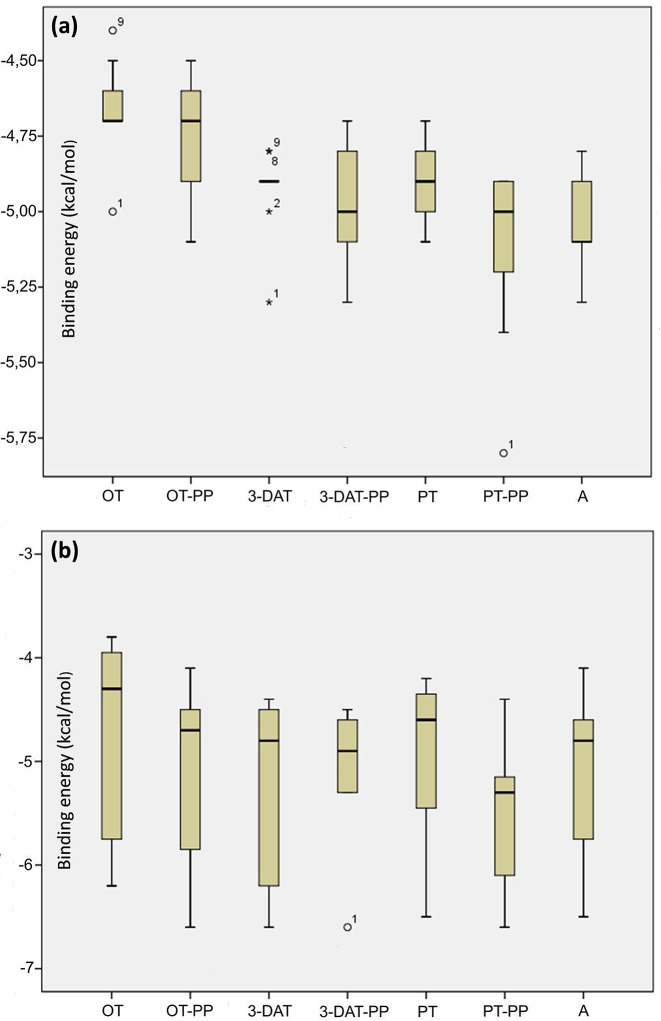
Statistical distribution of binding energies at binding points of thiamine and its derivatives to the pyruvate decarboxylase (**a**) binding of anti-coenzymes in the case of thiamine diphosphate already bound with active center, (**b**) binding of thiamine diphosphate in the case of anti-coenzyme already bound; 3-DAT, 3-deazathiamine; A, amprolium; OT, oxythiamine; PT, pyrithiamine; –PP, diphosphate esters of above mentioned compounds; Th, thiamine.

To take a closer look at the possible biological activity of the thiamine in comparison with corresponding anti-coenzymes, for the purpose of this work, we utilized the structure-based computer-aided chemical compound design simulations, which have undoubtedly made significant impacts to the drug development process [115,109]. Our research focused on predicting both the end-point of the ligand binding process (lowest-energy binding pose of a ligand and its corresponding binding energy) and statistical description of other poses, which reflect the diversification of possible binding sites. Chemical compound binding and unbinding are transient processes which are hardly observed by experiment and difficult to analyze by computational techniques. Toward this end, various docking methods were developed and continually improved to perform virtual screening of compound libraries for optimization. In this work, we used the Autodock Vina program [127], which implements an iterated local search with global optimization method using an empirical scoring function, which method is applicable to finding docking pathway for all types of binding sites from surface docking positions to interior ones. This methodology, successfully used previously by many research groups (see e.g. [128,129]), was applied to find the binding affinity between enzymes (yeast pyruvate decarboxylase PDC (RCSB PDB: 1QPB) and transketolase from *E. coli* TKT (RCSB PDB: 1QGD)) and diphosphate derivatives of thiamine and its analogs. As it is seen in Table 1, the affinity (both minimum and average) of all tested anti-coenzymes is comparable to that of thiamine diphosphate. Among the ligands, pyrithiamine shows the lowest binding energy, which makes it the most efficient anti-coenzyme. The affinity of 3-deazathiamine is significantly smaller, actually smallest over the whole set, which is in opposite to the hypothesis of extremely inhibiting strength of this ligand. These conclusions are further supported by the detailed statistical analysis for docking to PDC (Figure 10). The medians for all ligands are quite similar (i.e*.* within 1 kcal/mol) and no specific ligand can be chosen as “favorited”. To take a closer look at the hypothesis pictured in Figure 9, we also performed simulations of simultaneous docking of coenzyme and anti-coenzyme to the active site of the yeast PDC. The results are pictured in Figure 11. Four distinct situations are possible: significant excess of thiamine (only coenzyme is docking), partial activity (a minor excess of anti-coenzyme docking to the active site with thiamine diphosphate already bound), partial inhibition (a minor excess of coenzyme docking to the active site with anti-coenzyme already bound) and, finally, complete inhibition (significant excess of anti-coenzyme, only anti-coenzyme is docking). It is seen in Figure 11 that both partial activity as well as partial inhibition correspond to similar median of binding energies (∼ −4.5 kcal/mol), which is, however, significantly higher than these corresponding to normal activity (only coenzyme docking) and total inhibition (only anti-coenzyme docking).

**Table 1:** Minimum and average (over the nine best poses/binding sites combinations) binding energies for docking of thiamine, thiamine antivitamins, and phosphate derivatives.

	**PDC docking**		**TKT docking**		**TPK docking**		
	Minimum docking energy (kcal/mol)	Average docking energy (kcal/mol)	Minimum docking energy (kcal/mol)	Average docking energy (kcal/mol)	Minimum docking energy (kcal/mol)	Average docking energy (kcal/mol)	
**Thiamine P–P**	−8.9	−7.4	–10.7	−10.6	−7	−6.4	**Thiamine**
**Oxythiamine P–P**	−8.3	−6.8	−6.9	−6.2	−6.5	−5.9	**Oxythiamine**
**3-Deazathiamine P–P**	−7.7	−6.7	−7.5	−6.2	−7.2	−6.3	**3-Deazathiamine**
**Pyrithiamine P–P**	−9.8	−7.8	−10	−8.1	−7.1	−7.1	**Pyrithiamine**
					−7.1	−6.7	**Amprolium**

Shortcuts: PDC, subunit of yeast pyruvate decarboxylase (RCSB PDB: 1QPB); TKT, transketolase from *E. coli* TKT (RCSB PDB: 1QGD); TPK, thiamine pyrophosphokinase from yeast (RCSB PDB: 1IG0). Symbol P–P means diphosphate.

**Figure 12:**
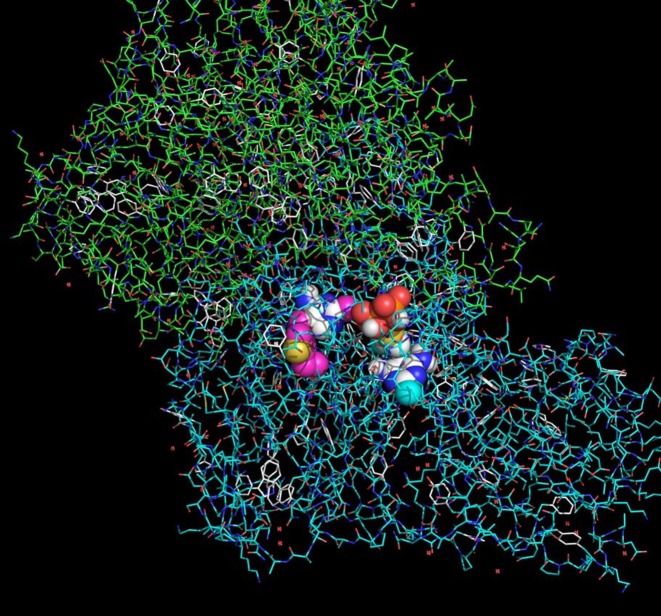
Molecule of 3-deazathiamine blocking the active center of the pyruvate decarboxylase. Subunit of pyruvate decarboxylase is shown as sticks, 3-deazathiamine (on left) and thiamine diphosphate (on right) are shown as spheres.

The largest (although still not significant) difference between partial activity and partial inhibition is observed for amprolium, where partial binding energy is lowest. This may suggest that amprolium, behaving differently with excess of coenzyme/anti-coenzyme, is powerful inhibitor of thiamine-dependent reactions although it does not form diphosphate derivatives. We found only a few binding poses to oxythiamine and its diphosphate. This observation may suggest that docking of this ligand is very selective, which may also limit its anti-coenzyme activity against pyruvate decarboxylase. The process of interaction of thiamine diphosphate and 3-deazathiamine in the subunit of yeast PDC (1QPB) is depicted in Figure 12. 3-Deazathiamine molecule blocks the active center of the enzyme, thus preventing thiamine diphosphate from proper (i.e. with lower energy as listed in Table 1) binding. The binding energy, corresponding to the mentioned above “mixing” docking (≈4.5 kcal/mol, Figure 11), is significantly lower than for “pure” binding (≈8 kcal/mol, Table 1 and Figure 10), without anti-coenzyme already bound. From the above considerations, we can agree that the processes suggested on the Figure 9 are computationally possible and may affect the proper thiamine diphosphate binding to the enzyme active site. However, partially inhibited enzymes are not as stable as “pure” thiamine diphosphate and thiamine antivitamins complexes.

Another enzyme which uses thiamine as a substrate for phosphorylation process to form thiamine diphosphate is thiamine pyrophosphokinase. This enzyme could use thiamine antimetabolites like oxythiamine or pyrithiamine as substrates to form their diphosphate esters [130,131,132]. This process can inhibit thiamine diphosphate synthesis in the cell. Most potent inhibitor of thiamine pyrophosphokinase among mentioned thiamine antivitamins is pyrithiamine (inhibition constant, 2–3 μM) in comparison with oxythiamine (4.2 mM). Although amprolium, which is not able to form diphosphate derivatives, can also inhibit thiamine pyrophosphokinase (inhibition constant, 180 μM [133]). For the sake of comparison, we also performed simulations of molecular docking of thiamine and related compounds as subtracts to thiamine pyrophosphokinase from yeast (RCSB PDB: 1IG0). Results, shown in the right part of Table 1, indicate that the affinity of thiamine is very similar to that shown by antivitamins, which supports the conclusion that the active site of the enzyme can be effectively blocked by all tested thiamine antimetabolites. However, these results do not support the previously mentioned hypothesis of extreme inhibiting efficiency of 3-deazathiamine.

### Thiamine antivitamins in animal models, cell cultures, and tumors

*In vivo* experiments carried out on rats confirmed results of *in vitro* enzymological research. Response of thiamine diphosphate-dependent enzymes on thiamine antivitamins in rats was dose related. Low doses (0.5 μM of oxythiamine/100 g body weight, every 12 h, up to 20 injections) caused inhibition of TK after 16 injections and PDHC after 12 injections. OGDHC was resistant to oxythiamine administration during all the time of experiment [122]. In contrast, high dose of oxythiamine (1 mM/kg body weight, single injection) caused more than 4-fold decrease in OGDHC as well as PDHC activities in adrenal mitochondria after 2–4 h [134]. In that experimental conditions, higher sensitivity on oxythiamine show OGDHC while inhibition of TK occurred later. Oxythiamine administration also caused inhibition of some thiamine diphosphate-independent enzymes like 6-phosphogluconate dehydrogenase and NADP-dependent malate dehydrogenase [134]. Similarly, pyrithiamine treatment (5 μg/10 g mice body weight daily up to 10 days) in combination with thiamine-deficient diet despite inhibition of PDHC (10%) and OGDHC (21%) caused decrease in thiamine diphosphate-independent succinate dehydrogenase (27%) and succinate thiokinase (24%) activities. These results indicated that thiamine antivitamins could cause oxidative stress which affects efficiency of all Krebs cycle reactions [135], which may have significant consequences for whole bioenergetics of the cell.

In mammals pyrithiamine, in contrast with oxythiamine, crosses blood–brain barrier [131]. Pyrithiamine, oxythiamine, and amprolium reduce thiamine transport into the brain, enhanced thiamine diphosphate dephosphorylation, and lead to reduction in total thiamine level [136]. Despite of influence on thiamine transport, oxythiamine strongly decreases TK activity in different tissues of rats but not in brain [137]. In contrast, pyrithiamine affects TK and OGDHC in thalamus by decrease in mRNA level [138,139]. Moreover, it reduces the number of neurons and increases frequency of microglia cells in mice nerve tissue [140]. Taking into consideration these results, pyrithiamine is used to induce thiamine deficiency-like status [36,79,141] in animal models and to understand how thiamine deficiency affects the functioning of the nervous system [37,142].

There are experimental data that thiamine antagonists like amprolium, oxythiamine, or pyrithiamine caused apoptosis of rat pheochromocytoma PC-12 cells. All these thiamine antagonists trigger apoptosis by mitochondria-dependent caspase 3-mediated signaling pathway. Pyrithiamine and oxythiamine display higher potency of apoptose induction than amprolium [143]. Additionally, it has been shown that amprolium inhibits PDHC by limiting the concentration of thiamine diphosphate and causes significant decrease in the concentration of acetyl-CoA during *in vitro* culture of cholinergic murine neuroblastoma cells [144].

Referring to above cited results, thiamine antivitamins were studied as potential tumor cell growth inhibitors. Inhibition of TK by thiamine antivitamins is expected to decrease the amount of ribose-5-phosphate which is needed for nucleic acid synthesis and cell proliferation. High decrease in tumor cells proliferation in Ehrlich’s tumor hosting mice and Mia pancreatic adenocarcinoma *in vitro* after administration of oxythiamine was observed [145,146]. This effect was related to inhibition of pentose phosphate pathway by decrease in TK activity. The cells were arrested in G_1_ phase of the cell cycle similar to the result of 2-deoxyglucose treatment. Administration of oxythiamine in combination with dehydroepiandrosterone sulfate - an inhibitor of glucose-6-phosphate dehydrogenase - (0.5 μM each) resulted in 60% inhibition of tumor cell proliferation *in vitro. In vivo* treatment of mice with 400 mg/kg body weight of oxythiamine caused more than 90% decrease in the Ehrlich’s tumor mass after 3 days of treatment. The histotoxicity analysis of liver, heart, and kidney of mice after oxythiamine treatment shows no signs of toxicity in comparison with control animals [147,145]. Another results show that oxythiamine treatment of normal human fibroblasts (30–1000 μM for 24–48 h) did not affect their viability and caused increase in collagen synthesis [148]. These results show that inhibitors of ribose synthesis like thiamine antivitamins could be considered as anticancer drugs. On the other hand, N3′-pyridyl thiamine (another antagonist of thiamine) almost completely suppresses activity of TK in HTC-116 tumor cells *in vivo* and *in vitro* but simultaneously did not affect OGDHC activity. In this case, despite of transketolase inhibition, there was no apparent effect on tumor cell growth [35]. This result indicated that inhibition of other thiamine diphosphate-dependent enzymes besides TK may be important in the limitation of tumor cell proliferation.

During analysis of oxythiamine action on thyroid tumor cells, a weak effect on thymidine uptake and expression of glucose transporter GLUT1 as well as transketolase isoenzyme TKTL-1 expression was shown. Therefore, oxythiamine cannot be generally recommended for the treatment of TKTL-1 expressing thyroid tumors [149]. On the other hand, recent data concerning docking of oxythiamine to the protein show that this antivitamin could be a potent inhibitor of human TKTL-1 [80].

Inhibition of Mia pancreatic carcinoma cell proliferation by oxythiamine was accompanied by loss of the activity of Hsp27 which is related to cancer cell survival. Oxythiamine caused increase in tumor cells in G_1_/G_0_ phase simultaneously reduces the number of cells in G_2_/M phase by suppression of expression of CDK4 and cyclin D1 [146]. This effect can be related to inhibition of transketolase which causes global deficit of nucleotides. Together with inhibition of OGDHC and PDHC, it causes a deficiency of high-energy phosphate bounds (ATP and GTP) resulting in decrease in proteins phosphorylation, for example Hsp27. The results obtained using Mia pancreatic carcinoma cell indicate that interference of oxythiamine on thiamine diphosphate-dependent enzymes altered multiple cellular signaling pathways associated with promotion of cell apoptosis [150].

During the investigation of growth and metastasis of Lewis lung carcinoma, it was shown that oxythiamine inhibited cell invasion and migration *in vitro* (IC_50_ = 8.75 μM). Mice treatment with high (500 mg/kg body weigh) or low (250 mg/kg body weigh) dose daily for 5 weeks caused decrease in plasma metalloproteinases (MMP-2 and MPP-9) activity and increased expression of tissue inhibitors of metalloproteinase (TIMP-1 and TIMP-2) [151]. Observed effects may be in relation to oxythiamine influence on thiamine diphosphate-dependent enzymes which restriction may be the major mechanism of this anticancer effect [43]. Degradation of extracellular matrix by metalloproteinase and its increased expression are associated with tumor cell invasion. Therefore, demonstrated oxythiamine action is very beneficial in cancer therapy especially due to antimetastatic efficacy.

Other data [152,109] indicate that oxythiamine can be useful in therapy of drug resistance cancer. Combination of oxythiamine (transketolase inhibitor) with dehydroepiandrosterone (glucose-6-phosphate dehydrogenase inhibitor) was effective in arresting metatrexate-resistant cancer cell proliferation (human colon adrenocarcinoma M6-HT29). The effectiveness of that treatment show that there are more than one effective way to inhibit ribonucleic acid synthesis, what is critical for cancer cell survival. Combined therapy using oxythiamine and imatinib (tyrosine kinase inhibitor used in the treatment of chronic myeliod leukemia) led to reduction of *in vitro* growth of imatinib-resistant tumor and enhanced the efficacy of imatinib in primary chronic myeloid leukemia isolated from patients. Probably use of oxythiamine or other thiamine antivitamins which inhibit TK, PDHC, and OGDHC can enhance cytostatics efficiency of other known anticancer drugs.

### Thiamine antivitamins impact on parasites and microorganisms

Coccidiosis is the diseases that contracts breeding animals and is a common cause of diarrhea and weight loss. It is caused by a protozoa parasite from genus *Eimeria*. Amprolium is good and widely used anticoccidiosis agent which effectively reduces the level of fecal *Eimeria* oocysts in cattle and poultry [153,6]. It is administered orally, often as feed additive, in a dose of 30–50 mg/kg body weight, leading to blood plasma concentration approximately 50 µg/ml. Toxic dose of amprolium (600 mg/kg) induces cerebrocortical necrosis in animals [154–156]. Recently, large amounts of veterinary drugs are used around the world and risk of food and environmental contamination generates the need for search on simple methods for detecting such contaminants as amprolium in food products [154,157] as well as their impact on environment [56].

Pyrithiamine was shown to be toxic in small amounts to fungi and bacteria. In the case of yeast, pyrithiamine and oxythiamine inhibit growth rate but when these two analogs were added to the medium together no growth inhibition occurred. This phenomenon was explained by thiamine synthesis from pyrimidine moiety of pirythiamine and thiazolium moiety of oxythiamine [158].

In the case of yeast cultured 3 days on medium with 40 mg/l of oxythiamine, an increase in pyruvate decarboxylase activity was observed. Simultaneously, oxythiamine decreased both the growth rate and survival ability of yeast [159]. This unusual effect may be the result of earlier inhibition of PDC which causes an accumulation of pyruvate while mitochondrial PDHC and OGDC were inhibited by oxythiamine at the same time [160]. Accumulation of pyruvate and inhibition of PDHC and OGDHC may cause increased biosynthesis of PDC apoform which was activated by endogenous thiamine diphosphate. At the same time, activity of transketolase was unchanged. These results suggest that decrease in growth rate of yeast caused by oxythiamine may be the result of mitochondrial enzymes inhibition and down-regulation of Krebs cycle and ATP synthesis by oxidative phosphorylation. These data are in accordance with other results showing that *Malassezia pachydermatis*, an opportunistic aerobic pathogen of dogs and cats which is associated with *otitis externa*, is more sensitive to oxythiamine (MIC = 1.25 − 2.5 μg/ml) in comparison with *Candida* and filamentous fungi (MIC > 160 μg/ml) which can provide fermentative as well as oxidative metabolism [161]. Oxythiamine also affects the lipid metabolism of *Saccharomyces cerevisiae, Candida albicans*, and *M. pachydermatis* in a different manner. In the case of *M. pachydermatis* grown on the medium with oxythiamine, total fatty acid content decreases approximately 50% in comparison with control [162]. The results of our recent studies also point to the practical potential of thiamine antivitamins, especially oxythiamine. We have found that this thiamine derivative has cytostatic effect against *M. pachydermatis* [44]. In addition, we have shown an synergistic effect of oxythiamine and commonly used antifungal agent—ketoconazole. The combination of these two compounds led to reduction of the effective concentration against *M. pachydermatis* by several orders of magnitude in comparison with each of the compounds acting alone. These studies were the basis for the patent application [163].

Many data indicate that pyrithiamine and oxythiamine action on microbes and fungi is additionally mediated by interaction with riboswitches. Phosphate esters of thiamine analogs bind to the riboswitch with a stretched conformation of thiazolium and pyrimidine ring of oxythiamine diphosphate as well as pyridine and pyrimidine ring of pyrithiamine diphosphate causing down-regulation of expression of *thiM* and *thiC* genes involved in thiamine *de novo* biosynthesis [57,39,164,113]. As riboswitches are generally not present in mammals and humans, they can serve as very efficient and effective antibacterial and antifungal drug targets.

In conclusion, oxythiamine and other thiamine antivitamins could be consider as a useful surfactant in the therapy of superficial mycoses, especially caused by species which cannot provide fermentative metabolism like *Malassezia* [44].

## Outlook

In the light of current knowledge of the role of thiamine in cell metabolism, we can assess the effects of its deficiency as well as the mechanisms and effects of thiamine antimetabolites on our organisms. As a result of this knowledge, both thiamine and its antimetabolites are becoming increasingly use in medicine and veterinary practice. Thiamine used as a dietary supplement is important for improving the well being of older people, especially those affected by neurodegenerative diseases. It is recommended by geriatrics, neurologists, and cardiologists to use it in appropriate doses and easily absorbed form (such as benfotiamine). Therefore, research on the process of absorption of thiamine and finding its well-absorbed forms as well as defining of groups of risk of thiamine deficiency become a great interest of medical doctors.

Taking into consideration the above mentioned results of many studies, thiamine antivitamins could be considered as useful additional agents in the therapy of cancer, superficial mycoses (especially these caused by species which cannot provide fermentative metabolism like *Malassezia*), and bacterial infections. Introducing new therapies is very important in terms of bacteria and fungi increasing drug resistance. From this point of view, synthesis of new thiamine derivatives based on strong thiamine diphosphate-dependent enzymes inhibitors is very interesting scientific task. The use of new theoretical and organic chemistry tools provides opportunities for the design and synthesis of compounds with desirable affinity to target proteins in the cell. Comparing the effects of new derivatives with known anticoenzymes on the level of thiamine diphosphate-dependent enzymes and pathogenic yeast, bacteria and cancer cells, we can estimate the utility of obtained derivatives and show perspectives for their practical use in medicine.

On the other hand, recent research of Zhang et al. [132] indicates that we can be exposed to trace amounts of thiamine antimetabolites like oxythiamine as a result of thiamine transformation through cooking under acidic conditions at 100°C. That kind of contamination may cause undesirable effects on our metabolism (e.g. transketolase inhibition in dialyzed patients with end-stage renal disease). Poultry fed with amprolium as a means of preventing coccidiosis as well as post-production impurities from poultry farms may be also potential sources of thiamine antimetabolites contamination. From this point of view, there is a need for intensive development of new methods for the measurements of thiamine antimetabolites in food, feedstocks, and environment in order to constant monitoring of the level of contamination and prediction of the possible effects of thiamine antimetabolits pollution for people health.

## Abbreviations

HIF1α: hypoxia inhibitory factor α1
OGDHC: 2-oxoglutarate dehydrogenase complex
PDC: pyruvate decarboxylase
PDHC: pyruvate dehydrogenase complex
TK: transketolase
